# Circ-EPB41L5 regulates the host gene *EPB41L5* via sponging miR-19a to repress glioblastoma tumorigenesis

**DOI:** 10.18632/aging.102617

**Published:** 2020-01-06

**Authors:** Tao Lv, Yifeng Miao, Tianqi Xu, Wenhua Sun, Youzhou Sang, Feng Jia, Xiaohua Zhang

**Affiliations:** 1 Department of Neurosurgery, Ren Ji Hospital, School of Medicine, Shanghai Jiao Tong University, Shanghai 200127, China; 2 Department of Neurosurgery, Ren Ji Hospital South Campus, School of Medicine, Shanghai Jiao Tong University, Shanghai 201112, China; 3 State Key Laboratory of Oncogenes and Related Genes, Renji-Med X Clinical Stem Cell Research Center, Ren Ji Hospital, School of Medicine, Shanghai Jiao Tong University, Shanghai 200127, China

**Keywords:** glioblastoma, circ-EPB41L5, miR-19a, EPB41L5, AKT

## Abstract

Background: Circular RNAs (circRNAs) are widely expressed non-coding RNAs in eukaryotic cells, involved in regulating tumorigenesis of several types of cancers. However, the expression profiles and the precise functional role in glioblastoma remain unclear.

Results: Circ-EPB41L5 was downregulated in glioblastoma tissues and cell lines compared to the normal brain tissues and cell lines. Low circ-EPB41L5 expression was correlated to the poor prognosis of glioblastoma patients, while the overexpression inhibited proliferation, clone formation, migration, and invasion abilities of glioma cells, and the suppression had counter effects. Furthermore, RNA-seq results determined that the host gene was the target gene of circ-EPB41L5, which served as a sponge against miR-19a and inhibited miR-19a activity from upregulating the expression of EPB41L5. Finally, we found that circ-EPB41L5 regulated the RhoC expression and phosphorylation of AKT through EPB41L5.

Conclusion: The current study highlights a novel suppressive function of circ-EPB41L5 and reveals that circ-EPB41L5/miR-19a/EPB41L5/p-AKT regulatory axis plays a striking role in the progression of glioblastoma, which provides a novel insight into the mechanisms underlying glioblastoma.

Methods: The expression profiles of circRNAs in glioblastoma were determined by Illumina HiSeq from six glioblastoma tissues and six normal brain tissues. Then, the correlation between circ-EPB41L5 expression and clinical features and the survival time of 45 glioblastoma patients was detected. The interaction between circ-EPB41L5, miR-19a, and EPB41L5 was assessed by luciferase reporter and RNA pull-down assays. The effects of expression of the ectopic intervention of circ-EPB41L5 or EPB41L5 on proliferation, clone formation, migration, and invasion in vitro and tumorigenesis in vivo were observed to evaluate the function of circ-EPB41L5 or EPB41L5.

## INTRODUCTION

Glioblastoma is the most common primary aggressive malignant brain tumor of the central nervous system [1]. Currently, maximum feasible surgical resection followed by radiotherapy plus concomitant and adjuvant temozolomide chemotherapy is the primary treatment for glioblastoma [2]. Despite great advances in target therapy, immunotherapy, and biotherapy, most treatments are less standardized and have not revealed significant improvements in the survival, and the prognosis of glioblastoma patients remains poor with a median overall survival of approximately 15 months [3, 4]. The developments in next-generation sequencing have revealed the characteristic genetic and epigenetic profiles of glioma [5–9]. Recent studies have identified that molecular biomarkers refine tumor diagnostics and improve the prediction of treatment response and outcome [10–12]. The WHO classification of central nervous system tumors of 2016 considers the altered molecular genetics and employs a new diagnostic concept [13]. Therefore, further study of the complex gene regulation network and novel treatment approaches to glioblastoma is an urgent requisite.

circRNAs were identified as non-coding RNAs in eukaryotic cells about 40 years ago and misinterpreted as the by-product of aberrant RNA splicing for the past few decades [14]. With the advances in high-throughput sequencing and bioinformatics, the circRNAs have been deemed as stable, conserved, abundant, and involved in physiological and pathophysiological processes of various diseases, including cancers [15–17]. Several core functions of circRNAs have been revealed: microRNA (miRNAs) sponges, regulators of transcription and splicing, binders to RNA-binding proteins, and translation into peptides. miRNAs are non-coding RNAs (ncRNAs) with a highly conserved gene expression regulator that effectuates via binding to the 3′-untranslated region (3′-UTR) of the target mRNAs [18]. Cumulative studies have reported that circRNAs, lncRNAs, and pseudogenes can bind to miRNAs to inhibit their activity and further regulate the downstream gene expression [19, 20]. Long non-coding RNAs (lncRNAs) acting as miRNA sponge have been confirmed in different cancers, including glioblastoma [21, 22]. However, the expression profile and potential function of circRNAs in glioblastoma are yet to be elucidated.

In this study, we detected the expression profile of circRNAs in glioblastoma and identified a circular RNA, termed as circ-EPB41L5, derived from the *EPB41L5* gene, which was significantly downregulated in glioblastoma tissues and cell lines and associated with the prognosis of glioblastoma patients. Furthermore, in vitro and in vivo experiments found that circ-EPB41L5 affected the proliferation, migration, and invasion abilities of glioblastoma. Supposedly, circ-EPB41L5 functioned as a sponge of miR-19a to regulate the expression of its host gene *EPB41L5* according to the bioinformatics method and confirmed by the luciferase reporter, RNA pulldown, and biotin-miRNA pulldown assays. These findings imply that circ-EPB41L5 may be a novel glioblastoma-suppressor circRNA, which can serve as a potent potential biomarker and therapeutic target for glioblastoma.

## RESULTS

### Dysregulated expression of circRNAs in glioblastoma

**Figure 1:**
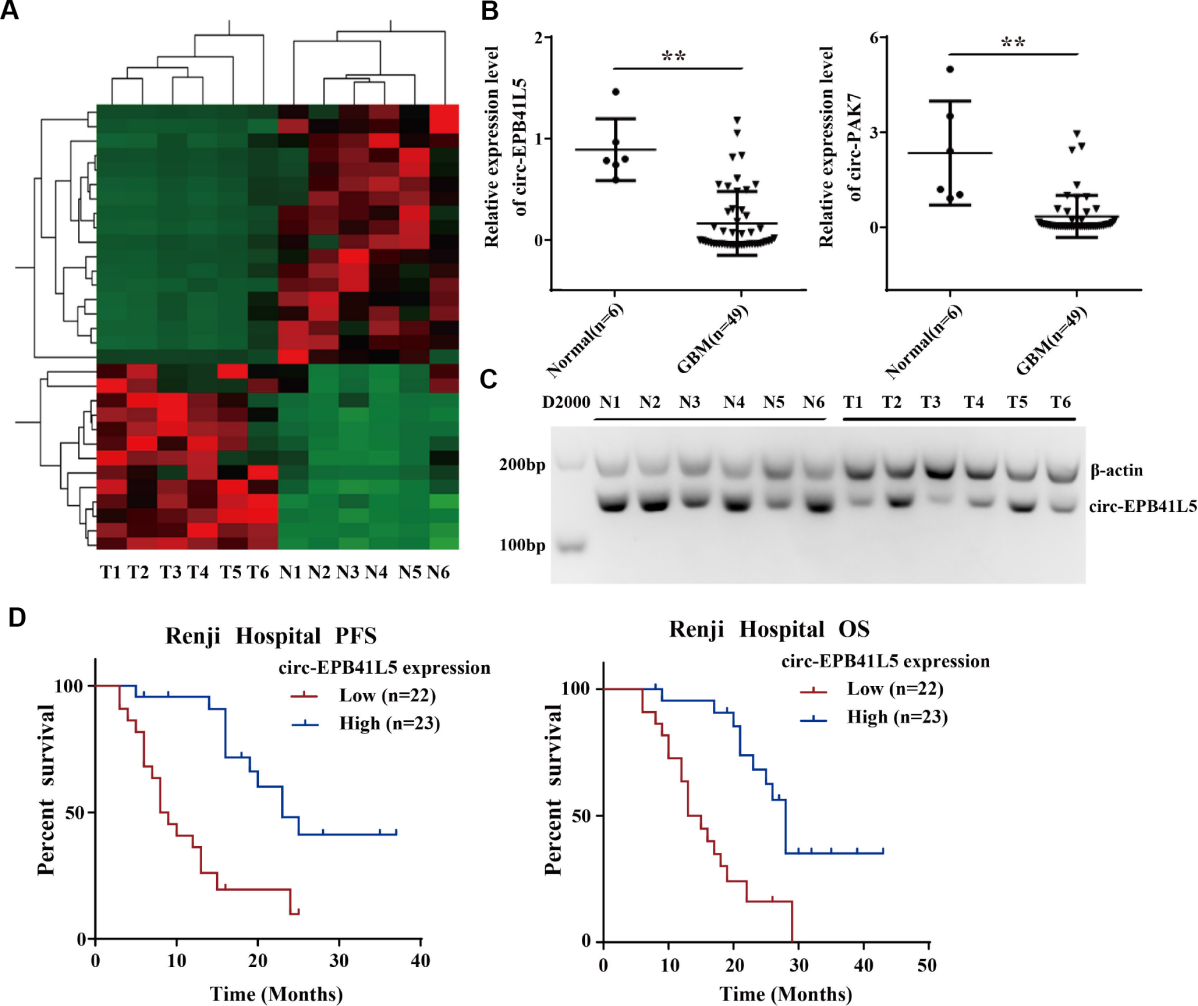
**Profile of circRNAs in human glioblastoma samples and circ-EPB41L5 characterization.** (**A**) Hierarchical cluster analysis of RNA-seq data was used to present the significantly dysregulated circRNAs between a normal brain and glioblastoma tissues. Red represents high expression, and green represents low expression. (FC≥2.0, *P*<0.05, FDR<0.05). (**B**) circ-EPB41L5 and circ-PAK7 were downregulated in glioblastoma detected by qRT-PCR (49 Glioblastoma and 6 normal). (**C**) RT-PCR gels showed that circ-EPB41L5 were downregulated in glioblastoma. (**D**) Prognostic significance of circ-EPB41L5 expression for glioblastoma patients. The expression median value was used as the cutoff. *P<0.05, **P<0.01.

The ribosomal RNA-depleted total RNA was used to generate the RNA-seq database of circRNAs. A total of 22454 circRNAs identified in six glioblastoma tissues and six normal brain tissues were identified (Supplementary Figure 1A). The median length of circRNAs was 700 nt (Supplementary Figure 1B). The results revealed that most of the host genes generated multiple circRNAs (Supplementary Figure 1C). Hierarchical clustering and volcano plot showed differentially expressed circRNAs, including 13 upregulated circRNAs and 18 downregulated circRNAs (FC≥2.0, *P*<0.05, FDR<0.05, Figure 1A). The dysregulated circRNAs are listed in Supplementary Table 3. GO analysis demonstrated that the host gene of dysregulated circRNAs were involved in various biological process: synaptic transmission, mitotic nuclear division, chromosome segregation, microtubule polymerization, and mitotic cell cycle (Supplementary Figure 1D). Then, qRT-PCR was conducted to verify the expression of five candidate dysregulated circRNAs (circRNA-EPB41L5, circRNA-PAK7, circRNA-NEK4, circRNA-STK33, circRNA-SYNE2) in six normal brain tissues and 49 glioblastoma tissues. As shown in Figure 1B, circ-EPB41L5 and circ-PAK7 were significantly downregulated in glioblastoma tissues. Subsequently, the expression and function of circ-EPB41L5 were investigated. Analysis of RT-PCR products by electrophoresis revealed the expression of circ-EPB41L5 in six glioblastoma and normal brain tissues (Figure 1C), which was consistent with the qRT-PCR results. The Kaplan–Meier analysis revealed that glioblastoma patients with low circ-EPB41L5 expression had significantly poorer progression-free survival (PFS) and overall survival (OS) as compared to those with high circ-EPB41L5 expression (*P*<0.001 for both; Figure 1D).

### Clinical significance of circ-EPB41L5 in glioblastoma

To evaluate the clinical significance of circ-EPB41L5, we detected its expression in 45 glioblastoma patients whose clinical characteristics were known. The results demonstrated that the expression of circ-EPB41L5 was associated with age (*P*=0.026), number of lesions (*P*=0.03), necrosis change (*P*=0.03), recurrence (*P*=0.031), and survival (*P*=0.038) (Table 1). However, circ-EPB41L5 was not associated with sex, Karnofsky performance status (KPS) score (preoperative), tumor size, and edema. Furthermore, the univariate analysis suggested that low expression of circ-EPB41L5, elder age, lower preoperative KPS, multiple lesions, and recurrence were closely associated with the poor PFS and OS (Table 2). In addition, multivariate Cox analysis showed that low expression of circ-EPB41L5 was an independent factor for poor PFS and OS in glioblastoma patients (hazard ratio (HR)=3.408, 95% confidence interval (CI): 1.318–8.397; HR=3.401, 95% CI: 1.388–8.880, respectively) (Table 3). Taken together, low expression of circ-EPB41L5 presented a poor prognosis, acting as a putative suppressor in glioblastoma.

**Table 1:** Correlation between circRNA-EPB41L5 expression and clinical features in human GBM.

**Characteristics**		**Total**	**Relative expression of circRNA-EPB41L5**		
			**Low**	High*	***P* Value†**
Sex					
	Male	24	13	11	0.449
	Female	21	9	12	
Age					
	≤65	24	8	16	0.026*
	>65	21	14	7	
KPS score (preoperative)					
	≤70	13	8	5	0.279
	>70	32	14	18	
No. of lesions					
	Single	34	13	21	0.03*
	Multiple	11	9	2	
Tumor size (cm3)					
	≤10	17	7	10	0.42
	>10	28	15	13	
Necrosis Change					
	With	20	11	9	0.03*
	Without	25	11	14	
Edema					
	With	32	18	14	0.22
	Without	13	4	9	
Recurrence					
	Yes	31	19	12	0.031*
	No	14	3	11	
Survival					
	Yes	16	4	12	0.038*
	No	29	18	11	

* The median expression level of circRNA-*EPB41L5* was used as the cutoff.

^†^ Pearson’s chi-square tests were used to analyse the correlation between circRNA-*EPB41L5* expression and clinical features, results were considered statistically significant at *P* <0.05.

**Table 2:** Univariate analyses for the association between patient characteristics and PFS and OS in GBM.

**Characteristics**		**PFS**			**OS**		
		**1-year Rate (%)**	**2-year Rate (%)**	***P* Value†**	**1-year Rate (%)**	**2-year Rate (%)**	***P* Value†**
Sex	Male/Female	61.6 vs. 71.4	27.1 vs. 32.2	0.76	82.9 vs. 85.7	41.9 vs. 43.4	0.991
Age	≤60/>60	83.1 vs. 45.9	39.8 vs. 20.1	0.01*	91.3 vs. 65.5	59.2 vs. 19.4	0.002**
KPS score (preoperative)	≤70/>70	59.2 vs. 68.6	10.2 vs. 36.8	0.047*	83.9 vs. 84.3	0 vs. 52.9	0.041*
No. of lesions	Single/Multiple	73.1 vs. 45.5	46.4 vs. 0	0.01*	81.6 vs. 72.7	56.2 vs. 9.1	0.005**
Tumor size (cm3)	≤10/>10	76.0 vs. 59.7	42.2 vs. 20.4	0.302	87.7 vs. 74.4	45.6 vs. 46.1	0.37
Necrosis Change	With/Without	53.8 vs. 76.0	15.8 vs. 40.4	0.057	73.9 vs. 83.6	30.2 vs. 52.9	0.091
Edema	With/Without	58.6 vs. 84.6	35.9 vs. 23.7	0.531	74.4 vs. 91.7	42.8 vs. 42.3	0.773
Recurrence	Yes/No	61.3 vs. 77.9	17.0 vs. 57.7	0.022*	74.2 vs. 92.3	33.9 vs. 64.7	0.046*
Relative expression level of circRNA-EPB41L5‡	Low/High	36.4 vs. 95.7	9.7 vs. 48.2	<0.001**	72.7 vs. 95.5	16.0 vs. 68.3	<0.001**

‡ The median expression level of circRNA-EPB41L5 was used as the cutoff.

† Kaplan-Meier method was used to calculate the 1-year and 2-year survival rate and Logrank method was used for univariate analyses, results were considered statistically significant at P <0.05. *< 0.05, **< 0.01.

**Table 3:** Multivariate analyses of prognostic factors affecting PFS and OS.

**Factors**	**PFS**			**OS**		
	**HR**	**95%CI**	**P Value**	**HR**	**95%CI**	**P Value**
Age	0.331	0.119-0.914	0.033*	0.414	0.159-1.079	0.071
KPS score	0.747	0.259-2.516	0.59	0.976	0.350-2.720	0.964
No. of lesions	0.457	0.173-1.207	0.114	0.644	0.242-1.709	0.376
Recurrence	1.671	0.575-4.857	0.346	1.294	0.443-3.779	0.638
Relative expression level of circRNA-EPB41L5	3.405	1.318-8.397	0.008**	3.401	1.388-8.880	0.007**

Results were considered statistically significant at P <0.05. *< 0.05, **< 0.01.

### Circ-EPB41L5 plays a suppressive role in glioblastoma cells in vitro

**Figure 2:**
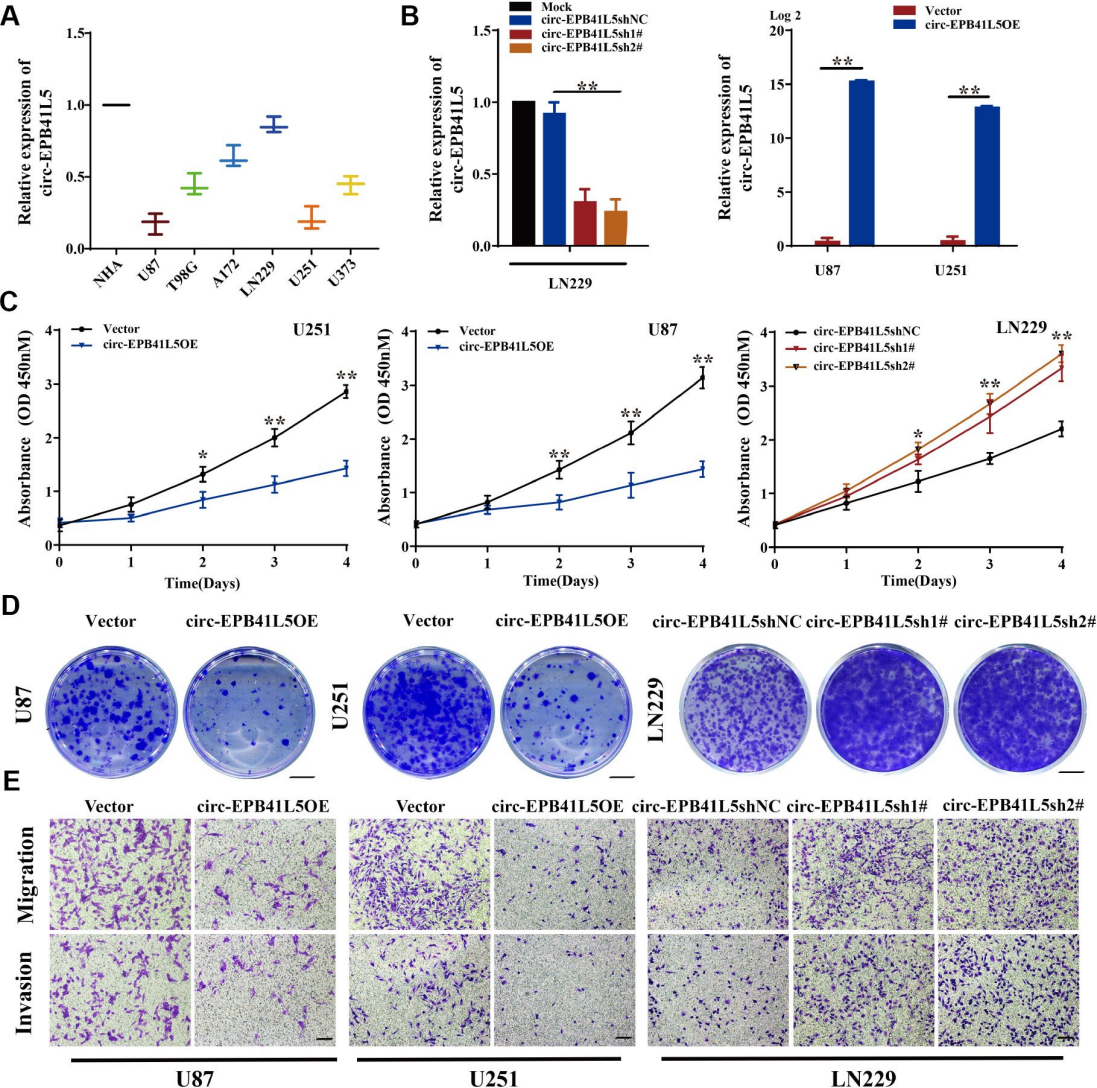
**Suppression effects of circ-EPB41L5 on glioma cells.** (**A**) qRT-PCR detected circ-EPB41L5 expression in normal human astrocytes (NHAs) and glioma cells (U87, T98, A172, LN229, U251, U373). (**B**) qRT-PCR tested the efficiency of circ-EPB41L5sh plasmids in LN229 cells. qRT-PCR tested the efficiency of circ-EPB41L5 overexpression plasmids in U87 and U251 cells. (**C**) CCK-8 assays determined the cell viability after stable transfection with vector, circ-EPB41L5sh, or circ-EPB41L5 overexpression plasmid in U87, U251, and LN229 cells. (**D**) Colony formation assays detected the cell proliferation ability; representative images are presented. Colony formation rates were normalized to the vector group. Scale bars: 4 mm. (**E**) Transwell assays measured the migration and invasion abilities of glioma cells; representative images are presented. Scale bars: 100 μm. The data are the means±SEM of three experiments, *P<0.05, **P<0.01.

circ-EPB41L5 was derived from exons 17–25 of the host gene *EPB41L5*; the mature sequence length was 962 bp (Supplementary Figure 2A). To evaluate the role of circ-EPB41L5 in glioblastoma, we detected its expression in several glioblastoma cell lines and found that circ-EPB41L5 is downregulated in glioblastoma cells, especially in U87 and U251, as compared to the normal human astrocytes (NHA) (Figure 2A). Then, the overexpression plasmid of circ-EPB41L5 was constructed in U87 and U251 (Figure 2B), and two shRNAs against the back-splicing of circ-EPB41L5 to silence the expression were designed in LN229 (Figure 2B). The CCK-8 assay showed that increased expression of circ-EPB41L5 repressed the proliferation of U87 and U251 cells (Figure 2C). Conversely, the silencing of circ-EPB41L5 promoted the cell proliferation in LN229 (Figure 2C). Furthermore, the overexpression of circ-EPB41L5, while silencing of circ-EPB41L5 improved the ability of colony formation (Figure 2D, Supplementary Figure 2B). Cell migration and invasion assays showed that the overexpression of circ-EPB41L5 impaired the migration and invasion abilities of glioma cells, and the downregulation presented an opposite role (Figure 2E, Supplementary Figure 2C).

### RNA-seq reveals that circ-EPB41L5 is targeted on the host gene *EPB41L5*

**Figure 3:**
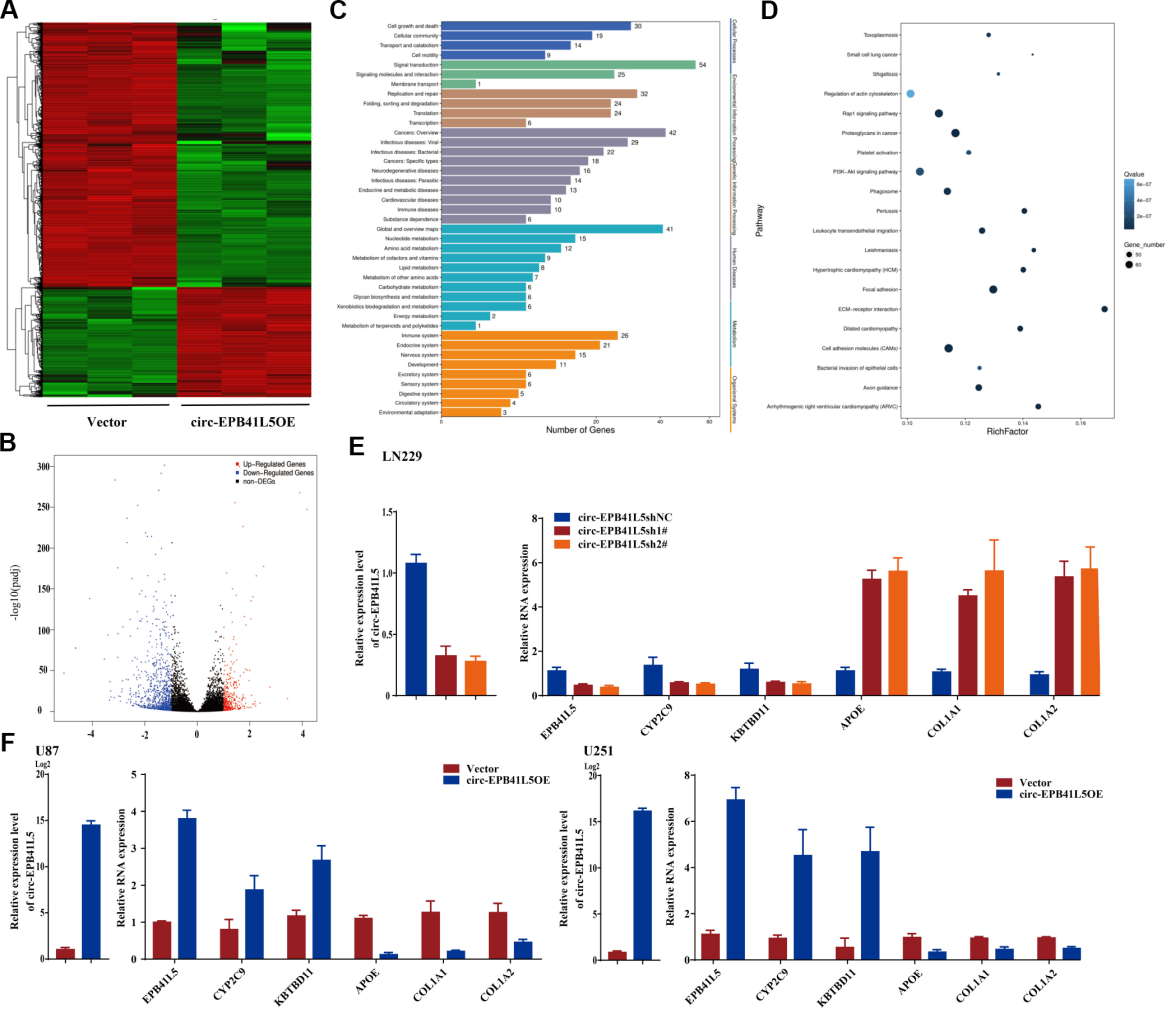
**RNA-seq analyzed target genes of circ-EPB41L5.** (**A**) Hierarchical cluster presented the significantly dysregulated genes after overexpression of circ-EPB41L5. Red represents high expression, and green represents low expression. (**B**) Volcano plot filtered out the dysregulated genes (FC≥2.0, *P*<0.05, FDR<0.05). Red and blue dots denote upregulated and downregulated genes, respectively. (**C**) KEGG analysis of upregulated genes. (**D**) Pathway enrichment analysis of downregulated genes. (**E**, **F**) qRT-PCR validated the dysregulated genes after overexpression or knockdown of circ-EPB41L5 in U87, U251, and LN229 cells.

RNA-seq was performed to investigate the putative mechanism by which circ-EPB41L5 affects the development of glioblastoma. After overexpression of circ-EPB41L5, 1349 dysregulated genes included 396 upregulated genes and 953 downregulated genes (FC≥2.0, *P*<0.05, FDR<0.05). The dysregulated genes are shown in the hierarchical clustering and volcano plot (Figure 3A, 3B). KEGG analysis revealed that upregulated genes are involved in the cell growth and death, cellular community, and cell motility (Figure 3C), while pathway enrichment showed that upregulated genes were involved in mismatch repair, DNA replication, and cell cycle (Supplementary Figure 3). Interestingly, the downregulated genes were found to be involved in Rap1, PI3K-Akt, and cell adhesion molecules that are crucial for tumorigenesis, and these results supported our hypothesis that circ-EPB41L5 acts as a suppressor in glioblastoma (Figure 3D). Consecutively, we found that the expression of *EPB41l5* mRNA was significantly increased in circ-EPB41L5 overexpressed glioma cells. Then, after the knockdown or overexpression of circ-EPB41L5 in U87, U251, and LN229, qRT-PCR was performed to detect the expression of the three upregulated genes (*EPB41L5*, *CYP2C9*, *KBTBD11*) and three downregulated genes (*APOE*, *COL1A1*, *COL1A2*) to verify the RNA-seq results. The data consisted of the RNA-seq of EPB41L5 obtained by specific primers (Figure 3E, 3F; Supplementary Figure 4).

### Circ-EPB41L5 suppresses glioblastoma depending on EPB41L5

**Figure 4:**
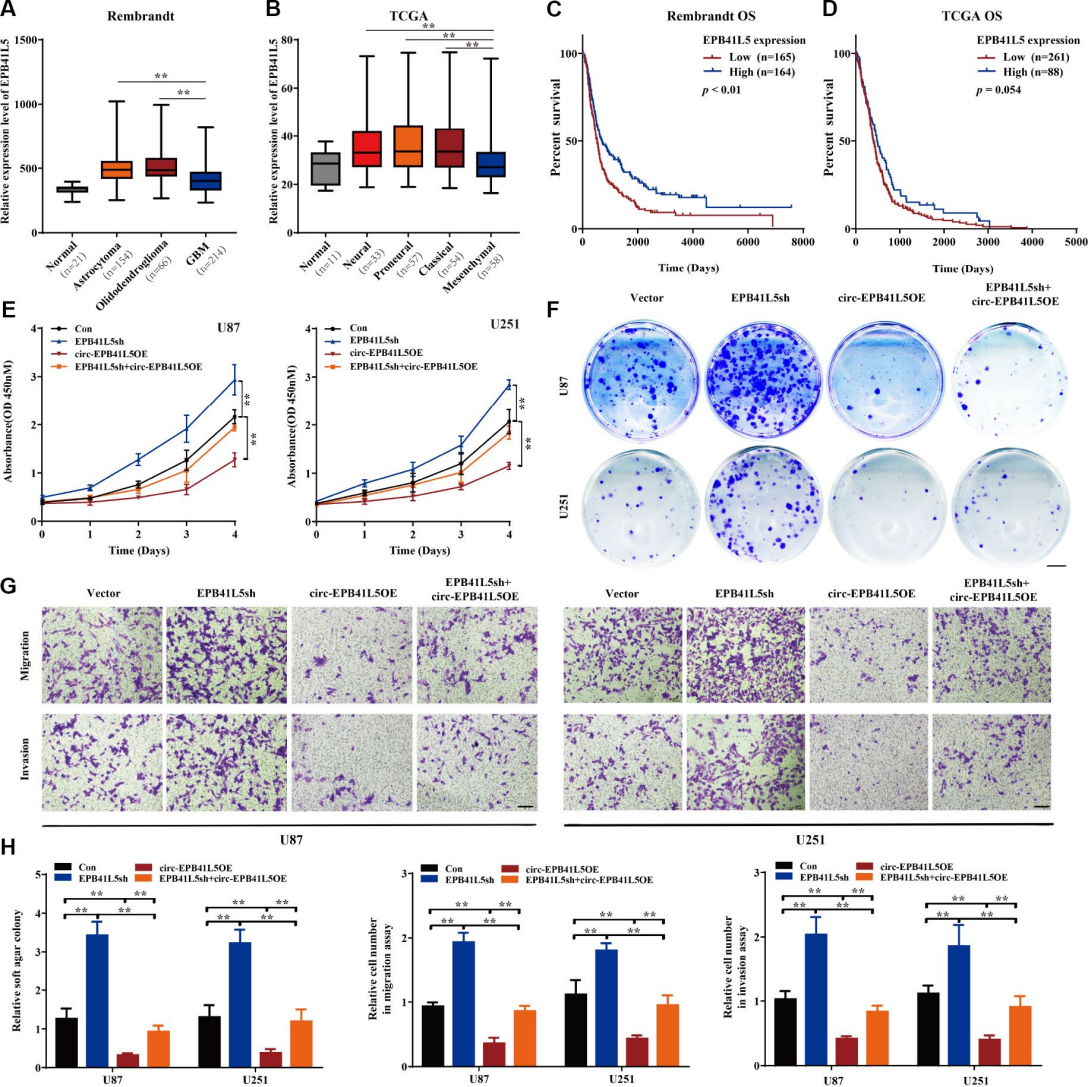
**circ-EPB41L5 suppressed glioma cell proliferation, migration, and invasion.** (**A**) The expression level of EPB41L5 in glioma from the Rembrandt dataset. The level of EPB41L5 was decreased in glioblastoma. (**B**) Expression level of EPB41L5 in different subtypes of glioblastoma from TCGA dataset. The level of EPB41L5 was the lowest in mesenchymal. (**C**, **D**) Kaplan–Meier survival analysis of glioblastoma patients from the Rembrandt and TCGA datasets. Low expression of EPB41L5 indicated poor prognosis. (**E**–**G**) Knockdown of EPB41L5 in U87 and U251 promoted proliferation, colony formation, migration, and invasion abilities of glioma cells. Overexpression of circ-EPB41L5 impaired the oncogenic effects of EPB41L5 knockdown. Scale bars: 4 mm in colony formation assays. Scale bars: 100 μm in transwell assays. (**H**) Quantification of colony formation rates and relative migration or invasion cell number. The data are means±SEM of three experiments, *P<0.05, **P<0.01.

In order to assess the function of EPB41L5 in glioblastoma, we first downloaded Rembrandt and TCGA datasets of glioma samples. In the Rembrandt dataset, 21 normal brain samples, 154 astrocytoma, 66 oligodendroglioma, and 214 glioblastoma were included. The expression of EPB41L5 was significantly elevated in glioma tissues as compared to normal samples. However, the level of EPB41L5 in glioblastoma was markedly lower than in astrocytoma and oligodendroglioma (Figure 4A). In the TCGA dataset, 11 normal, 33 neural, 57 proneural, 54 classical, and 58 mesenchymal samples were included. The expression of EPB41L5 in different subtypes of glioblastoma was significantly increased as compared to the normal samples. Interestingly, the level of EPB41L5 in the mesenchymal samples, the most malignant subtype, was the lowest (Figure 4B). Kaplan–Meier survival analysis in Rembrandt and TCGA datasets showed that glioma patients with low expression of EPB41L5 had a poor prognosis (Figure 4C, 4D). The above results suggested that EPB41L5 might also act as a suppressor in glioblastoma. Then, EPB41L5 was knocked down in U87 and U251, and the results showed that the abilities of proliferation, colony formation, migration, and invasion were improved. Simultaneously, the overexpression of circ-EPB41L5 could impair the effects caused by the knock-down of EPB41L5 (Figure 4E–4H).

### Circ-EPB41L5 regulates EPB41L5 expression via directly binding to miR-19a

**Figure 5:**
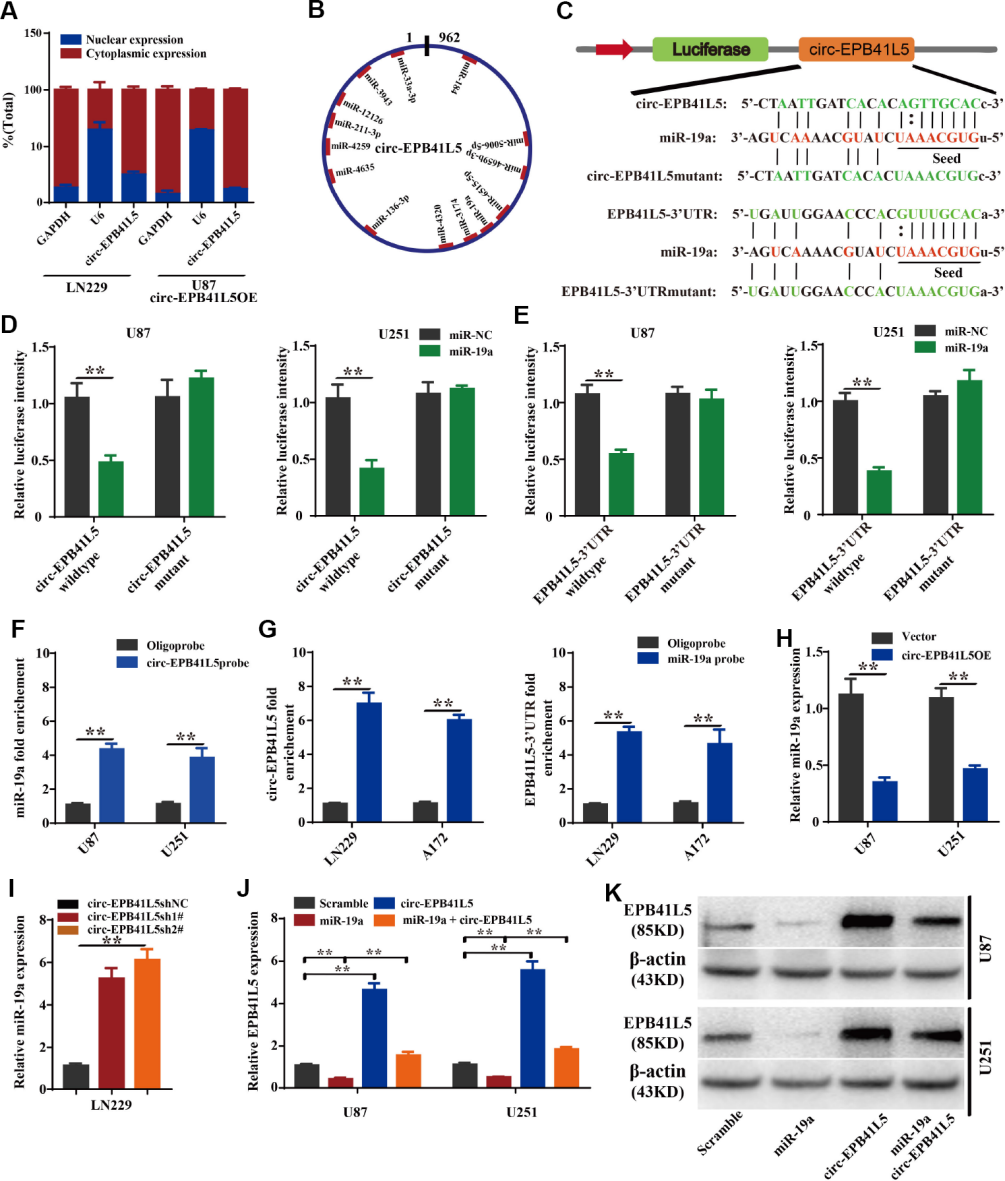
**circ-EPB41L5 functions as a sponge of miR-19a to regulate the expression of EPB41L5.** (**A**) Nuclear cytoplasm separation assay compared the abundance of circ-EPB41L5 in the nucleus and cytoplasm. Fractionation of LN229 and U87 cells followed by qRT-PCR. U6 RNA acted as a positive control for gene expression. (**B**) Schematic representation of the predicted binding sites of miRNAs related to circ-EPB41L5. (**C**) Schematic representation of circ-EPB41L5 or EPB41L5 3’-UTR wild-type (WT) and mutant (Mut) luciferase reporter vectors and miR-19a binding sites. (**D**, **E**) The relative luciferase activities were detected after co-transfection with miR-NCs or miR-19a mimics and circ-EPB41L5 or EPB41L5 3′-UTR WT and Mut luciferase reporter vectors in U87 and U251 cells. (**F**) Lysates from U87 and U251 cells were subjected to biotinylation-circ-EPB41L5 pulldown assay; qRT-PCR detected the expression of *miR-19a*. (**G**) Lysates from A172 and LN229 cells were subjected to biotinylation-miR-19a pulldown assay; qRT-PCR detected the expression of circ-EPB41L5 or EPB41L5-3’UTR. (**H**, **I**) qRT-PCR detected the expression of *miR-19a* in glioma cells transfected with circ-EPB41L5sh or circ-EPB41L5 overexpression plasmids. (**J**, **K**) qRT-PCR and WB assays detected the expression of EPB41L5 in glioma cells transfected with miR-19a mimics or circ-EPB41L5 vector. The data are the means±SEM of three experiments, *P<0.05, **P<0.01.

To further investigate the mechanism by which circ-EPB41L5 regulates the expression of EPB41L5, we detected the cellular location of circ-EPB41L5 through the nuclear cytoplasm separation assay. The results showed that circ-EPB41L5 is mainly localized in the cytoplasm (Figure 5A). Then, TargetScan and miRBase were used to predict which miRNAs interacted with circ-EPB41L5 or the 3′-UTR of the host gene. Consequently, 13 miRNAs binding sites were identified for circ-EPB41L5 (Figure 5B), among which, miR-19a could also bind to the 3′-UTR of EPB41L5. Furthermore, circ-EPB41L5, EPB41L5 3′-UTRs, and the mutant expression luciferase vectors were constructed (Figure 5C) and transiently cotransfected along with miR-19a mimics into U87 and U251 cells. The luciferase activity assay demonstrated that circ-EPB41L5 and 3′-UTR of EPB41L5 was a target of miR-19a (Figure 5D, 5E). Additionally, RNA pulldown analysis showed that biotinylated probes against circ-EPB41L5 could pull down endogenous miR-19a (Figure 5F). Moreover, endogenous circ-EPB41L5 and 3′-UTR of EPB41L5 could be pulled down by the miR-19a probe (Figure 5G). The above results confirmed the direct interaction of circ-EPB41L5 and EPB41L5 with miR-19a. Furthermore, the overexpression of circ- EPB41L5 remarkably inhibited the level of miR-19a (Figure 5H), while the knockdown augmented the level (Figure 5I). In addition, the overexpression of miR-19a mimics significantly inhibited the expression of EPB41L5, and the overexpression of circ-EPB41L5 restored the expression of EPB41L5 (Figure 5J). Western blot showed consistent changes (Figure 5K). These results indicated that circ-EPB41L5 might act as a sponge for miR-19a to regulate the expression of EPB41L5.

### Circ-EPB41L5 inhibits tumorigenesis of glioblastoma through activated p-AKT

**Figure 6:**
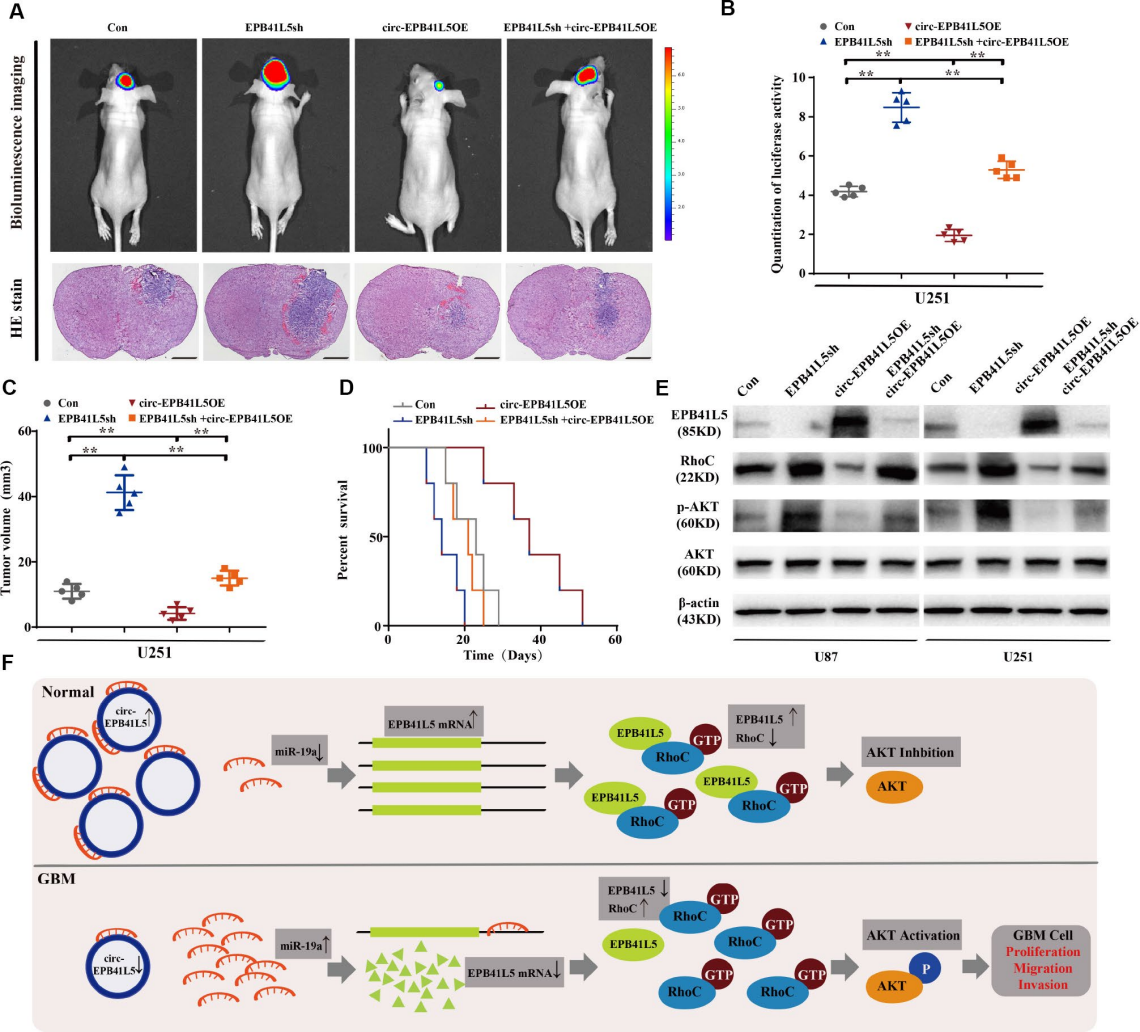
**Overexpression circ-EPB41L5 inhibited tumorigenesis of glioblastoma in vivo.** Overexpression circ-EPB41L5 inhibited the growth of brain xenograft tumors of U251 glioma cells. (**A**) Representative bioluminescence images and H&E stained images; scale bars: 1 mm. (**B**) Quantification of the bioluminescence activity. Data were from five mice per group. (**C**) Quantification of tumor size. Data were from five mice per group. (**D**) Kaplan–Meier survival analysis of mice implanted with U87 cells stably overexpressing circ-EPB41L5 or EPB41L5sh plasmids. Data were from five mice per group. (**E**) WB assays measured the expression of EPB41L5, RhoC, p-AKT, and total AKT in treated U87 and U251 cells. (**F**) Schematic representation of circ-EPB41L5-miR-19a-EPB41L5-AKT regulatory axis in glioblastoma. *P<0.05, **P<0.01.

We further investigated the effects of circ-EPB41L5 and its host gene on regulating the tumor growth in vivo. U251 cells were transfected with EPB41L5 knockdown or circ-EPB41L5 overexpression plasmids and stereotactically injected in nude mice. The results showed that knockdown EPB41L5 led to a marked increase in tumor volume with short survival time. Conversely, the overexpression of circ-EPB41L5 decreased the tumor formation in nude mice brain and impaired tumorigenesis caused by EPB41L5 knockdown. Additionally, mice implanted with overexpressed circ-EPB41L5 glioma cells showed maximal survival (Figure 6A–6C). Furthermore, Western blot was performed to verify the role of circ-EPB41L5/miR-19a/EPB41L5 regulatory axis on the downstream signaling pathways. Previous studies have proved that EPB41L5 directly interacts with and inhibits the activation of RhoC, which is a regulator of p-AKT [24–26]. The current results also indicated that the inhibition of EPB41L5 upregulated the levels of RhoC and p-Akt in U87 and U251, while the overexpression of circ-EPB41L5 downregulated the expression of RhoC and p-Akt and reversed the upregulation of RhoC and p-Akt mediated by EPB41L5 (Figure 6D). Taken together, circ-EPB41L5 affected the proliferation, migration, and invasion of glioma cells by regulating the miR-19a-EPB41L5, RhoC, and p-Akt signaling pathways (Figure 6E).

## DISCUSSION

Although the molecular mechanisms of glioblastoma have made progress, the prognosis of glioblastoma patients remains unfavorable due to the rapid proliferation and infiltration. Therefore, it is vital to study the new target molecules affecting the proliferation and invasion of glioblastoma. circRNAs are widespread RNA transcripts found in different species and diseases [27–29]. Numerous studies have proved that dysregulated expression profiles of circRNAs contribute to the initiation and promotion of different types of cancers [30, 31]. In this study, we identified the circRNA expression profile in glioblastoma, and 31 dysregulated circRNAs were observed. Subsequent experiments deduced that circ-EPB41L5 was significantly downregulated in glioblastoma tissues and associated with several clinical features, including age, number of lesions, necrosis change, recurrence, survival, and prognosis of glioblastoma patients. Functionally, the overexpression of circ-EPB41L5 inhibited the proliferation, migration, and invasion of glioma cells, which suggested that circ-EPB41L5 might play a suppressor role in the pathogenesis and development of glioblastoma.

Previous studies reported that circRNAs act as miRNA sponges, protein sponges, coding RNAs, or protein complex scaffolds for critical biological functions [32]. To date, several circRNAs have been reported to play crucial roles in gliomas, such as circ-SMARCA5, circ-MMP9, circ-NT5E, circ-TTBK2, and circ-LINC-PINT [33–37]. These studies proved that circMMP9, circNT5E, and circTTBK2 harbors the miRNAs to suppress the target genes in glioblastoma progression, circSMARCA5 directly interacts with SRSF1 to regulate the migration of glioma cells, and circ-LINC-PINT encodes a peptide to suppress the oncogenic transcriptional elongation in glioblastoma. In the current study, after the overexpression circ-EPB41L5, RNA-seq was performed to investigate the possible target genes and the downstream signaling pathways. Interestingly, EPB41L5 was regulated by circ-EPB41L5.

EPB41L5 (YMO1) is a member of the 4.1 protein family, which consists of eponymous 4.1R protein (EPB41), 4.1N (EPB41L1), 4.1G (EPB41L2), 4.1B (EPB41L3), NBL4 (EPB41L4A), and EHM2 (EPB41L4B) [38]. The 4.1 proteins can connect with the cell cortical cytoskeleton components, including transmembrane adhesion proteins, actin, spectrins, receptors, and transporters. Therefore, the 4.1 proteins are involved in several cellular process, such as the organization of cell polarity, cell adhesion, motility, and response to growth factors [39, 40]. The 4.1 proteins also contribute to the pathogenesis of several cancers, such as ovarian, hepatocellular, and glioma [41–43]. EPB41L5 interacts with cytoplasmic complex components via the C-terminal PDZ domain to regulate the epithelial cell architecture and polarity [44, 45]. Recent evidence indicates that EPB41L5 plays a major role in focal adhesion and differentiation of neurons [46, 47]. It has also been found that EPB41L5 suppresses the invasion and metastasis of hepatocellular carcinoma [26]. However, the role of EPB41L5 in glioma is unclear. Thus, we analyzed the expression of EPB41L5 in TCGA and Rembrandt databases and found that it was upregulated in the glioma but downregulated in glioblastoma as compared to LGG. Moreover, EPB41L5 was downregulated in mesenchymal glioblastoma that exhibits poor prognosis. In vitro and in vivo experiments demonstrated that the underexpression of EPB41L5 induced tumorigenesis of glioblastoma. Taken together, EPB41L5 is shown to act as a suppressor in the progression of glioblastoma.

miRNAs are small conserved regulatory non-coding RNAs. It has been demonstrated that miRNAs are involved in various biological functions in different diseases [48]. In addition, the circRNA-miRNA-mRNA axis serves as a widespread gene expression regulatory pattern [49]. In the present study, we found that circ-EPB41L5 was mainly localized in the cytoplasm, directly interacted with miR-19a, and inhibited its activity from regulating the expression of EPB41L5. miR-19a increases in glioma and is involved in the tumorigenesis of glioblastoma [50, 51].

The previous studies have indicated that EPB41L5 interacts with RhoC to suppress its expression and activity [26]. RhoC belongs to Rho GTPases and plays a key role in various cancers. Also, it is a vital regulator of cell migration, proliferation, and apoptosis, and therefore, inhibition of RhoC results in suppressing the tumor invasion and metastasis [52, 53]. Previous studies have demonstrated that RhoC phosphorylates AKT via activating ROCK1, and phosphorylation of AKT plays a critical role in the development of glioma. The current study identified the existence of circ-EPB41L5/miR-19a/EPB41L5/RhoC/AKT regulatory axis. circ-EPB41L5 inhibited the proliferation, migration, and invasion of glioma cells by sponging miR-19a and regulating the expression of the host gene *EPB41L5* that suppressed the progression of glioma by inhibiting RhoC and p-AKT.

## CONCLUSIONS

In conclusion, we identified a dysregulated circRNAs profile in glioblastoma and a novel target circRNA, circ-EPB41L5, that serves as a suppressor in glioblastoma. Additionally, we found that *EPB41L5* was the target gene of circ-EPB41L5 through RNA-seq and regulated by the circ-EPB41L5/miR-19a axis. Finally, we demonstrated that circ-EPB41L5/miR-19a/EPB41L5 axis promotes the tumorigenesis of glioblastoma via activated RhoC and phosphorylated AKT. The data highlighted that circ-EPB41L5 functions as a tumor suppressor and provides solid evidence to understand glioblastoma tumorigenesis and identify the potential therapeutic molecular targets for the treatment of glioblastoma.

## MATERIALS AND METHODS

### Tissue samples and cell culture

A total of 49 fresh glioblastoma tissues and six normal brain tissues were collected from patients during the operation at Renji Hospital, School of Medicine, Shanghai Jiao Tong University (Shanghai, China). Histological and pathological diagnostics of glioblastoma were evaluated according to the 2007 WHO classification of tumors of the central nervous system (CNS) and confirmed by two experienced pathologists, respectively. All glioblastoma patients received neither chemotherapy nor radiotherapy before the surgery. The tissues were stored at −80 °C. This study was approved by the Ethics Committee of Renji Hospital (No. 2017-058), and informed consent was obtained from patients before collecting the samples. RNA expression profiles and associated clinical data of the glioma patients were downloaded from the TCGA data portal (https://tcga-data.nci.nih.gov/) and GEO database (https://www.ncbi.nlm.nih.gov/). Human glioma cell lines U87, T98G, A172, LN229, U251, and U373 were obtained from the Cell Bank of Chinese Academy of Sciences and cultured in DMEM medium (Gibco, NY, USA) supplemented with penicillin/streptomycin and 10% fetal bovine serum (Gibco). All the cells were cultured at 37 °C in a humidified atmosphere with 5% CO_2_.

### Whole transcriptome sequencing and expression profile analysis of circRNAs

Total RNA was isolated using TRIzol (Thermo Fisher Scientific, Carlsbad, CA, USA) according to the manufacturer’s protocol. The RNA quantification and quality were measured via the NanoDrop ND-2000 spectrophotometer (Agilent Inc., USA). Whole transcriptome sequencing was performed on Illumina HiSeq Sequencer according to the manufacturer’s instructions. circRNAs were predicted according to the ACFS pipeline, and after normalization of the raw data, the circRNAs in at least two samples were analyzed further. The standard of significantly differentially expressed circRNAs was as follows: fold change (FC) ≥2.0, *P*-value<0.05, and false discovery rate (FDR)<0.05.

### Quantitative real time-polymerase chain reaction (qRT-PCR)

The cDNA was synthesized by reverse transcription using the PrimeScript RT reagent kit (TaKaRa, Japan) according to the manufacturer’s protocols. The expression of circRNAs and mRNAs was measured by qRT-PCR using SYBR premix ExTaq (TaKaRa) on Applied Biosystems Step One Plus Real-Time PCR system. Primers were purchased from HuaGene Biotech (Shanghai, China) and listed in Supplementary Table 1. β-actin was used as an internal control for circ-EPB41L5 and *EPB41L5*, *GAPDH*, and *U6* mRNAs were endogenous control in the nucleoplasm separation experiment. The relative expression of the RNAs was determined using the ΔΔCt method. Each qRT-PCR analysis was carried out in three independent experiments.

### Construction of plasmids and cell transfection

Short hairpin RNAs (shRNAs) for targeting to the junction region of the *circ-EPB41L5* or *EPB41L5* mRNA were purchased from HuaGene Biotech and cloned into FUGW-H1-Syndecan shRNA vector (No. 40623), and the sequences of shRNAs are listed in Supplementary Table 2. The full-length circ-EPB41L5 was cloned into pcDNA3.1 (+) CircRNA Mini Vector (No.60648), purchased from Addgene to construct the circ-EPB41L5 overexpression plasmid. Glioma cell lines were transfected with shRNAs plasmids of circ-EPB41L5 and EPB41L5 or circ-EPB41L5 overexpression plasmid using Lipofectamine 3000 (Invitrogen, Carlsbad, CA, USA).

### Prediction of miRNA targets

The interaction between circ-EPB41L5 or 3’-UTR of EPB41L5 and miRNA was predicted with TargetScan (http://www.targetscan.org) or miRBase (http://www.mirbase.org).

### Luciferase reporter assay

For luciferase reporter assay, the wild-type and mutant sequences of circ-EPB41L5 or 3′-UTR of EPB41L5 were cloned into pGL3-basic vectors (Realgene, Nanjing, China).

Then, the wild-type and mutant pGL3-LUC-circ-EPB41L5 or pGL3-LUC- EPB41L5- 3′-UTR and miR-19a mimics or inhibitor were co-transfected to 293T cells. After 48 h of transfection, the cells were harvested to measure the luciferase activities using the dual-luciferase reporter kit (Promega, Madison, WI, USA). Relative luciferase activities were presented, and each experiment was conducted in triplicate.

### Biotin-labeled pull-down assay

Biotin-labeled pull-down assay was performed as described previously [23]. Briefly, biotinylated circ-EPB41L5 and miR-19a probes (GenePharma, Shanghai, China) were incubated with streptavidin Dynabeads (Invitrogen) at 25 °C for 2 h. Then, 1×10^7^ glioma cells were lysed and sonicated to harvest cell lysates, which were incubated with probe-coated beads at 4 °C overnight. The RNA/beads complexes were washed and eluted. The level of EPB41L5, circ-EPB41L5, and miR-19a in complexes was determined by qRT-PCR analysis. Each experiment was carried out in triplicate.

### Western blots

Western blot (WB) assay was performed as as described below. Tissue samples were collected and then stored at −80 °C. After western blotting and electrictransfered to polyvinylidene fluoride (PVDF), membranes were incubated with primary antibodies at 4°C overnight. After washed with TBST for three times, membranes were incubated with secondary antibody. Primary antibodies were against EPB41L5 (1:500 dilution, Santa Cruz, CA, USA), RhoC (1:1000 dilution, Santa Cruz), AKT (1:1000 dilution, Cell Signaling Technology, Beverly, MA, USA), phospho-AKT (1:1000 dilution, Cell Signaling Technology) and β-actin (1:3000 dilution, Sigma–Aldrich, St. Louis, MO, USA). HRP-conjugated secondary goat anti-mouse or goat anti-rabbit antibodies were used at 1:5000 dilution (Proteintech, USA). Western blot was analyzed with ImageJ version 1.46 software (National Institutes of Health, Bethesda, MD).

### Cell counting Kit-8 (CCK-8) and colony formation assays

For CCK-8 assay, 1×10^3^ glioma cells/well were seeded into 96-well plate. Then, 10 μL/well CCK-8 solution (Beyotime, Shanghai, China) was added to each well at 0, 24, 48, 72, and 96 h. The optical density (OD) values were measured at 450 nm using a microplate reader (Bio-Tek, Winooski, VT, USA). For colony formation assay, 0.5×10^3^ glioma cells/well were seeded into a 6-well plate and cultured for 14 days. The colonies were fixed in 100% methanol for 10 min and stained with 1% crystal violet solution for 20 min at room temperature. The images were captured using an Olympus SZX12 stereomicroscope, and the number of colonies in each well was counted.

### Cell migration and invasion assay

Cell migration and invasion abilities of glioma cells were evaluated through transwell assays. After transfection with plasmids for 48 h, the cells were starved for 6 h and harvested to seed in 24-well cell culture inserts (BD Biosciences) with an 8-μm pore membrane. The membrane was coated without Matrigel for migration and invasion assay. The migrated and invaded cells were stained with crystal violet and counted. Each experiment was carried out in triplicate.

### Mice model for tumorigenesis studies

All experimental animal protocols were approved by the Shanghai Jiao Tong University Institutional Animal Care and Use Committee (IACUC). 6–8-week-old athymic (Ncr nu/nu) female mice (SLAC, Shanghai, China) were used. 1×10^6^ glioma cells were implanted into the brain of the animals stereotactically. Bioluminescence imaging was performed via the IVIS Lumina imaging station (Caliper Life Sciences). When neuropathological symptoms appeared, the mice were sacrificed, and tumor volumes were measured as (W2×L)/2, W<L (W: width; L: length).

### Statistical analysis

GraphPad Prism version 7.0 (GraphPad Software Inc., San Diego, CA, USA) was used to perform the two-tailed Student’s t-test for unpaired samples or one-way analysis of variance (ANOVA) with Newman-Keuls posthoc test for multiple comparisons. Chi-square test or Fisher’s exact test was performed to analyze the relevant factors. Kaplan–Meier survival probability analysis was carried out using log-rank tests. Cox regression analysis was used for multivariate analysis of prognosis factors. All data were presented as the mean±SEM. The criterion for significance was set as *P<*0.05.

## Supplementary Material

Supplementary Figures

Supplementary Tables
