# Supplementary material for: Circ-EPB41L5 regulates the host gene EPB41L5 via sponging miR-19a to repress glioblastoma tumorigenesis
Source: Aging (Albany NY). 2020 Jan 6;12(1):318–39. doi: 10.18632/aging.102617 (PMC6977680; doi:10.18632/aging.102617)
Supplement: Supplementary Figures [file aging-12-102617-s001..pdf]

SUPPLEMENTARY FIGURES

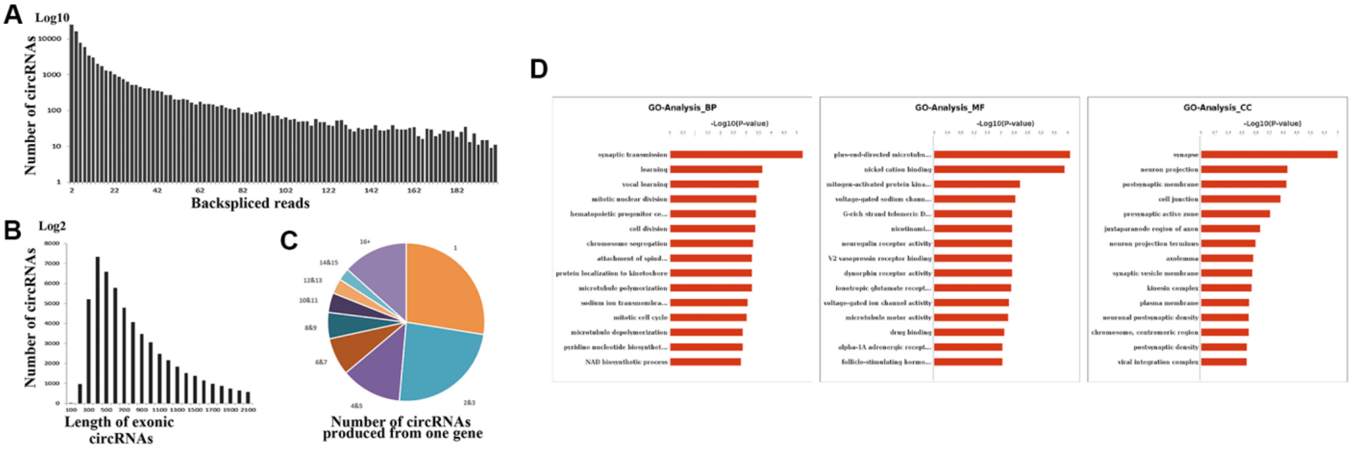

Supplementary Figure 1. (**A**) The back spliced reads and the corresponding number of circRNAs. (**B**) The length distribution for exonic circRNAs. (**C**) The number of circRNAs generated from one host gene. (**D**) GO analysis of the host gene of dysregulated circRNAs.

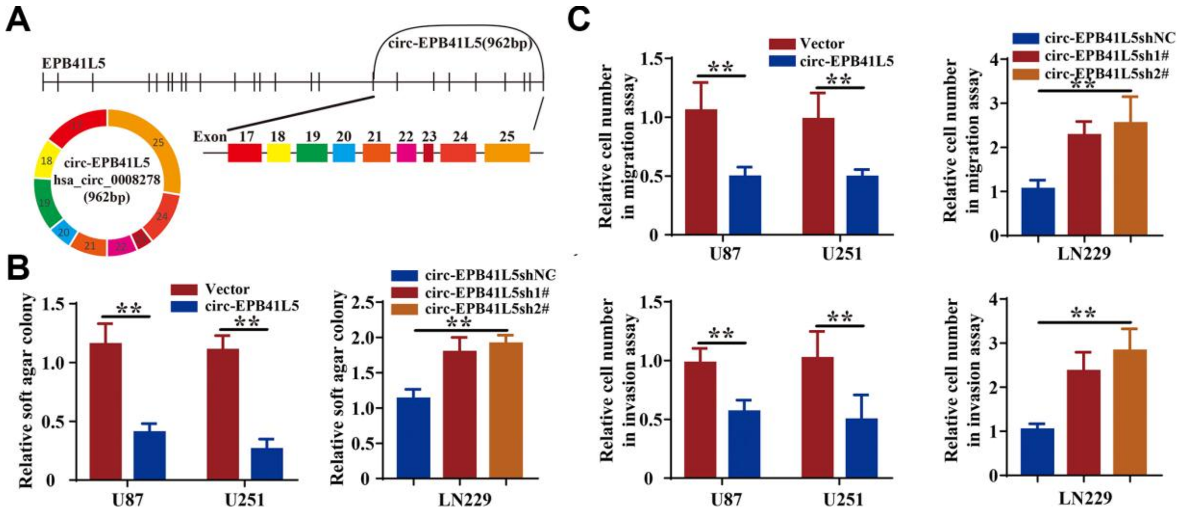

Supplementary Figure 2. (**A**) Schematic representation of circ-EPB41L5. (**B**) Quantification of Colony formation rates (**C**) Quantification of relative migration or invasion.

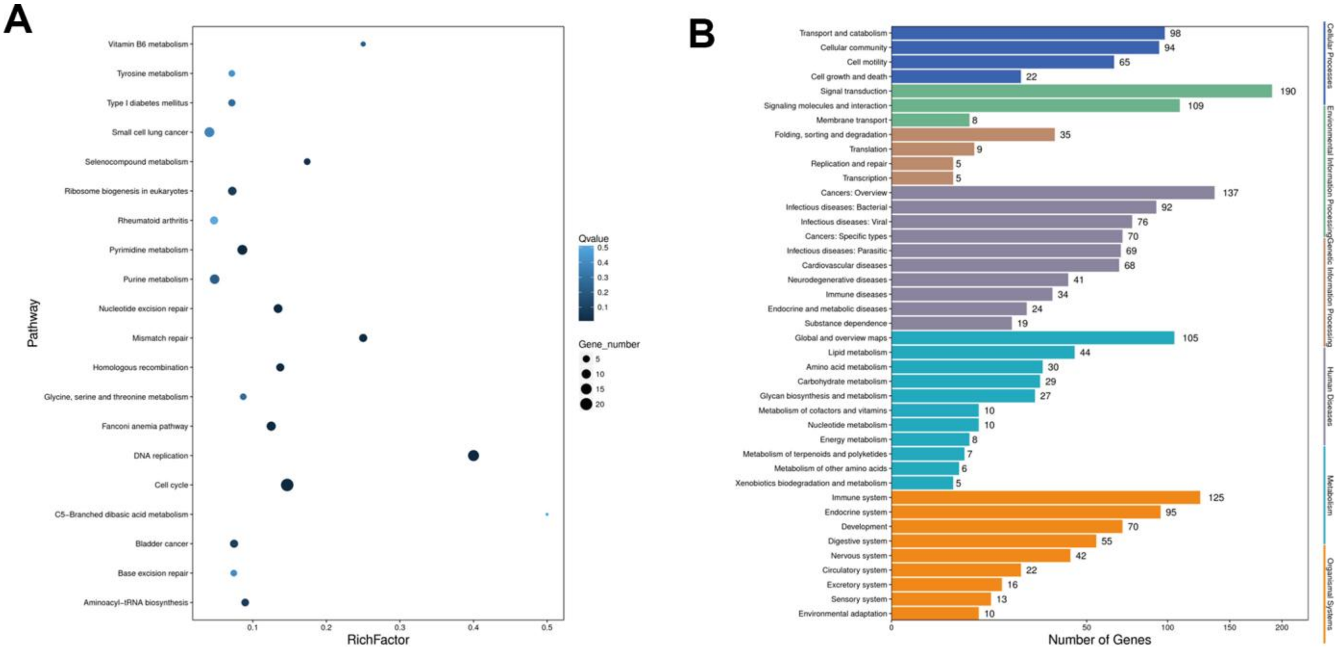

Supplementary Figure 3. (**A**) KEGG analysis of down-regulated genes. (**B**) Pathway enrichment analysis of up-regulated genes.

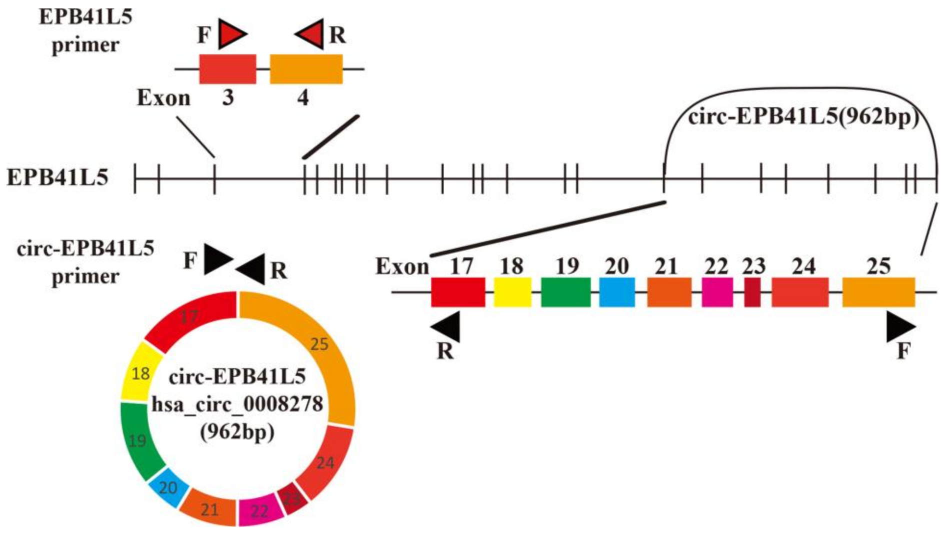

Supplementary Figure 4. The sketch of primers of circ-EPB41L5 and EPB41L5 mRNA. Red arrows represent primers amplifying genome region of EPB41L5 which does not contain circ-EPB41L5 sequence. Black arrows represent divergent primers, which are used to amplify the genome region of circPTK2 containing the back splice junction site.
