# Supplementary material for: Circ-EPB41L5 regulates the host gene EPB41L5 via sponging miR-19a to repress glioblastoma tumorigenesis
Source: Aging (Albany NY). 2020 Jan 6;12(1):318–39. doi: 10.18632/aging.102617 (PMC6977680; doi:10.18632/aging.102617)
Supplement: Supplementary Tables [file aging-12-102617-s002..pdf]

SUPPLEMENTARY TABLES

Supplementary Table 1. Real-time qRT-PCR primer list.

circRNAs	Primer sequences (5′-3′)
β-actin (Human)	F: CATGTACGTTGCTATCCAGGC
	R: CTCCTTAATGTCACGCACGAT
GAPDH (Human)	F: AGGGCTGCTTTTAACTCTGGT
	R: CCCCACTTGATTTTGGAGGGA
U6 (Human)	F: TGGCACCCAGCACAATGAA
	R: CTAAGTCATAGTCCGCCTAGAAGCA
circRNA-EPB41L5	F: AATACGCATCTCTCCAGCATTCC
	R: CCCAATTACATCACCAATCGTTGC
circRNA-PAK7	F: AGTGACTCCATCCTCCTGACAAG
	R: GCCCGATTTGCTTTGACTTTGAG
circRNA-NEK4	F: GGAGGAGGAGGATGAATTTGATAG
	R: CATTTGTCTGACCTTTCCCTTCC
circRNA-STK33	F: TGTCCCTGATGCCAATTACACTTC
	R: TGCCCACTTCGTTTCTGTTTCC
circRNA-SYNE2	F: GACTGGTTCAGCAACATTAAAGTG
	R: GCAGGTGGTGTTCAAGAATATCTC
mRNA-EPB41L5	F: TGTACCACCTGGACCTGATTGA
	R: ACCATCCAACCAATGTGCTACT
EPB41L5 3′UTR	F: CTCTCCAGCATTCCGTCCT
	R: GCAGTGTGATTCAACAATTCCA

F: forward; R: reverse

Supplementary Table 2. shRNAs for circRNA-EPB41L5.

RNAs	shRNAs (5′-3′)
sh-circRNA-EPB41L5-1	F:GATCCCCCATTATTTGTAGCATTCCTTTCAAGAGAAGGAATGCTACAAATAATGTTTTT<br>R:CTAGAAAAACATTATTTGTAGCATTCCTTCTCTTGAAAGGAATGCTACAAATAATGGGG
sh-circRNA-EPB41L5-2	F:GATCCCCGTCCATTATTTGTAGCATTTTCAAGAGAAATGCTACAAATAATGGACTTTTT<br>R:CTAGAAAAAGTCCATTATTTGTAGCATTTCTCTTGAAAATGCTACAAATAATGGACGGG
sh-EPB41L5	F:GATCCCCGGAGCTAACCCGGTATTTATTTTCAAGAGAAATAAATACCGGGTTAGCTCCTTTTT<br>R:CTAGAAAAAGGAGCTAACCCGGTATTTATTTCTCTTGAAAATAAATACCGGGTTAGCTCCGGG

Supplementary Table 3. The dysregulated circRNAs in glioblastoma.

CircRNAs	ACC-ID (Chrom_Start_End_ Length)	CircBase-ID	Regulation in GBM	p-Value	Log2 Fold Change	FDR	CircRNAs Type	Host Gene	SeqLength
circ_EPB41L5	chr2_120175004_120127688_47316	hsa_circ_0008278	down	4.10E-05	-4.53	3.94E-02	Exonic	EPB41L5	962
circ_ PAK7	chr20_9566384_9557608_8776	hsa_circ_0115623	down	2.37E-05	-5.83	3.94E-02	Exonic	PAK7	753
circ_ARHGAP44	chr17_12929046_12908897_20149	hsa_circ_0106205	down	1.44E-05	-20	3.16E-02	Exonic	ARHGAP44	384
circ_C10orf76	chr10_101995355_101975212_20143	hsa_circ_0003293	down	2.72E-05	-20	3.94E-02	Exonic	C10orf76	345
circ_DAB1	chr1_57023639_57010680_12959	——	down	6.88E-05	-20	4.86E-02	Intronic	DAB1	897
circ_ ELAVL2	chr9_23765099_23762006_3093	hsa_circ_0138518	down	6.15E-06	-20	2.05E-02	Exonic	ELAVL2	335
circ_ ERC1	chr12_1410453_1289852_120601	——	down	1.12E-06	-20	6.14E-03	Exonic	ERC1	470
circ_FBRS	chr16_30666541_30664215_2326	hsa_circ_0005806	down	5.73E-05	-5.14	4.49E-02	UnKnown	FBRS	2327
circ_GRM1	chr6_146399699_146398769_930	hsa_circ_0130947	down	3.45E-05	-20	3.94E-02	Intronic	GRM1	931
circ_ MAP7	chr6_136389517_136377755_11762	hsa_circ_0130788	down	4.97E-05	-20	4.04E-02	Exonic	MAP7	507
circ_ PCCA	chr13_100309908_100257595_52313	hsa_circ_0141073	down	3.40E-05	-20	3.94E-02	Exonic	PCCA	792
circ_RIMBP2	chr12_130422561_130399679_22882	hsa_circ_0097878	down	3.08E-05	-7.36	3.94E-02	Exonic	RIMBP2	771
circ_ RYR2	chr1_237469187_237369534_99653	hsa_circ_0112625	down	6.87E-05	-20	4.86E-02	Exonic	RYR2	1399
circ_ SLC4A10	chr2_161901011_161872293_28718	hsa_circ_0141742	down	4.37E-05	-20	3.94E-02	Exonic	SLC4A10	676
circ_ STX1A	chr7_73700931_73700378_553	hsa_circ_0009012	down	2.89E-05	-20	3.94E-02	Exonic	STX1A	309
circ_STXBP5L	chr3_121318540_121279805_38735	hsa_circ_0121601	down	1.96E-06	-20	8.58E-03	Exonic	STXBP5L	290
circ_ SYN3	chr22_33006824_32980645_26179	hsa_circ_0062997	down	1.95E-05	-20	3.88E-02	Exonic	SYN3	531
circ_ZNF337	chr20_25686466_25685567_899	——	down	3.70E-05	-20	3.94E-02	Exonic	ZNF337	299
circ_ EXOC6	chr10_92899644_92893349_6295	hsa_circ_0019170	up	2.23E-07	20	2.45E-03	Exonic	EXOC6	357
circ_FAM135A	chr6_70452571_70426439_26132	hsa_circ_0142354	up	3.88E-05	20	3.94E-02	Exonic	FAM135A	290
circ_ LCORL	chr4_17972885_17961903_10982	hsa_circ_0069285	up	6.10E-05	5.57	4.61E-02	Exonic	LCORL	276
circ_ MKLN1	chr7_131399433_131387120_12313	hsa_circ_0001747	up	9.82E-06	7.01	2.39E-02	Exonic	MKLN1	535
circ_ NEK4	chr3_52741499_52737586_3913	hsa_circ_0001309	up	4.50E-05	20	3.94E-02	Exonic	NEK4	429

circ_ POC1B	chr12_89472275_89466770_5505	hsa_circ_0027702	up	7.58E-06	20	2.08E-02	Exonic	POC1B	580
circ_POLE2	chr14_49674427_49663315_11112	hsa_circ_0008002	up	4.86E-05	20	4.04E-02	UnKnown	POLE2	510
circ_PTPRZ1	chr7_121984117_121972541_11576	hsa_circ_0133159	up	7.46E-07	20	5.45E-03	Exonic	PTPRZ1	624
circ_RIC8B	chr12_106825820_106783997_41823	——	up	4.47E-05	20	3.94E-02	Exonic	RIC8B	752
circ_ STK33	chr11_8464822_8413495_51327	hsa_circ_0096614	up	2.86E-05	6.29	3.94E-02	Exonic	STK33	1005
circ_SYNE2	chr14_64022863_63998914_23949	hsa_circ_0102377	up	6.54E-06	20	2.05E-02	Exonic	SYNE2	2284
circ_TDRD3	chr13_60460540_60439688_20852	——	up	1.90E-07	20	2.45E-03	Exonic	TDRD3	308
circ_ USP3	chr15_63574403_63553715_20688	hsa_circ_0035654	up	3.03E-05	20	3.94E-02	Exonic	USP3	812
